# Skeletal Muscle Gauge and Complementary Pan-Immune-Inflammation Value for Risk Stratification of 30-Day Major Complications After Colorectal Cancer Surgery

**DOI:** 10.3390/biomedicines14040894

**Published:** 2026-04-15

**Authors:** Botond-István Kiss, Daniela Tatiana Sala, Renáta Moriczi, Szabolcs-Attila Gábor, Árpád Török, Gabriel-Mircea Muresan, Tivadar Bara, Márton István Dénes, Szilárd-Leó Kiss, Szilárd-Leó Kiss, Orsolya Kiss-Toth, Radu-Mircea Neagoe

**Affiliations:** 1 Doctoral School of Medicine and Pharmacy, “George Emil Palade” University of Medicine, Pharmacy, Science and Technology of Târgu Mureș, 540139 Târgu Mureș, Romania; drkissbotondi@gmail.com (B.-I.K.); moriczi.renata@yahoo.com (R.M.); 2 2nd Department of Surgery, “George Emil Palade” University of Medicine, Pharmacy, Science and Technology of Târgu Mureș, 540139 Târgu Mureș, Romania; torokaea@gmail.com (Á.T.);; 3 2nd Clinic of Surgery, County Emergency Clinical Hospital, 540136 Târgu Mureș, Romania; gabor.szabi1@yahoo.com (S.-A.G.); mircea.muresan@umfst.ro (G.-M.M.);; 4 Department of Anatomy and Embryology, “George Emil Palade” University of Medicine, Pharmacy, Science and Technology of Târgu Mureș, 540139 Târgu Mureș, Romania; 5 1st Department of Gynecology and Obstetrics, “George Emil Palade” University of Medicine, Pharmacy, Science and Technology of Târgu Mureș, 540139 Târgu Mureș, Romania; 6 1st Clinic of Gynecology and Obstetrics, County Emergency Clinical Hospital, 540136 Târgu Mureș, Romania; 7 1st Clinic of Neonatology, County Emergency Clinical Hospital, 540136 Târgu Mureș, Romania

**Keywords:** sarcopenia, pan-immune-inflammation value, skeletal muscle gauge, 30-day major events, colorectal cancer, surgery

## Abstract

**Background**: Major morbidity and mortality remain important concerns after colorectal cancer (CRC) surgery. Cancer-related sarcopenia and heightened systemic inflammation may increase the risk of early postoperative major complications. **Methods**: In this retrospective single-center study, we analyzed 190 patients undergoing major CRC surgery. Skeletal muscle gauge (SMG) and the pan-immune-inflammation value (PIV) were assessed as preoperative risk markers, and 30-day major complications were evaluated. **Results**: Low SMG was strongly associated with major complications (OR 6.50, 95% CI 3.24–13.05; *p* < 0.001), and high PIV was also associated with increased risk (OR 3.51, 95% CI 1.77–6.99; *p* < 0.001). In multivariable analysis adjusting for age, surgical urgency, and procedure type, low SMG and emergency surgery remained independent predictors of 30-day major complications. The highest-risk phenotype (high PIV/low SMG; n = 23) had a major complication rate of 78.3% (18/23) (*p* < 0.001). A clinical model including age, urgency, and procedure type yielded an AUC of 0.739 (95% CI 0.661–0.816). Adding low SMG improved discrimination (AUC 0.784, 95% CI 0.711–0.857), with only a small additional increase after adding high PIV (AUC 0.791, 95% CI 0.717–0.864). **Conclusions**: Preoperative low SMG was independently associated with 30-day major complications after CRC surgery, while PIV provided complementary risk-stratification value. The combined high-PIV/low-SMG phenotype identified patients with particularly high postoperative risk.

## 1. Introduction

Colorectal cancer (CRC) remains a leading global health burden, ranking among the most frequently diagnosed malignancies and the major causes of cancer-related death worldwide [1]. Despite substantial advances in surgical technique, enhanced recovery pathways, and perioperative care, early (30-day) morbidity remains common after major CRC surgery and continues to drive prolonged hospitalization, readmissions, delayed adjuvant therapy, and excess healthcare costs [2,3,4]. Accurately identifying patients at highest risk is therefore central to perioperative decision-making, nutritional optimization, and appropriate allocation of resources, yet risk stratification based solely on conventional clinical variables is often insufficient [5].

In this context, increasing attention has been directed toward the host phenotype—particularly secondary sarcopenia and cancer-associated inflammation—as potentially modifiable determinants of postoperative outcomes [6,7]. Secondary sarcopenia in oncology reflects progressive skeletal muscle loss driven by catabolic metabolism, reduced anabolic signaling, decreased intake, and physical inactivity, ultimately impairing muscle mass, strength, and functional reserve [8,9]. CRC is frequently accompanied by systemic inflammation and immune dysregulation, which can accelerate muscle wasting and promote myosteatosis (fatty infiltration of skeletal muscle), a feature linked to reduced muscle quality and metabolic dysfunction [6,10,11]. While cancer cachexia represents a prototypical inflammatory syndrome underlying muscle and fat loss, secondary sarcopenia in CRC is multifactorial and may also be influenced by age-related vulnerability, tumor burden, treatment effects (e.g., chemotherapy), and the physiologic stress of surgery [8,9]. Skeletal muscle is the body’s largest protein reservoir and supplies amino acids during major surgical stress; in advanced sarcopenia, depleted stores may reduce the metabolic reserve needed for effective immune function and postoperative tissue repair [10,12]. These overlapping nutritional and inflammatory pathways provide a rationale for integrated biomarkers capturing both body composition and immune–inflammatory status [7,13].

Computed tomography (CT)-derived body composition analysis is widely considered a reference standard for preoperative evaluation of skeletal muscle because CT is routinely performed for CRC staging and enables objective quantification [14,15]. Beyond muscle quantity alone, composite metrics incorporating muscle quality may better reflect physiologic reserve [16,17]. Skeletal muscle gauge (SMG), which integrates muscle area and attenuation, provides a combined assessment of muscle quantity and quality and may offer a more clinically informative representation of sarcopenic phenotypes than single-parameter indices [18].

Complementing imaging-based assessment, inflammation-based biomarkers derived from routine blood tests have emerged as accessible indicators of the systemic host response [13]. The pan-immune-inflammation value (PIV), calculated as (neutrophils × platelets × monocytes)/lymphocytes, integrates multiple leukocyte and platelet components and has been explored across oncologic and inflammatory conditions [19]. By incorporating several immune cell lines, PIV may capture immune–inflammatory dynamics more comprehensively than simpler ratios and may be relevant for perioperative risk assessment in CRC [19,20].

Accordingly, this study aimed to evaluate preoperative SMG and PIV in relation to 30-day major complications after major CRC surgery, to quantify their discriminatory performance, and to determine whether a combined SMG–PIV phenotype identifies a subgroup with particularly high postoperative risk. Such an approach may support improved perioperative risk assessment, patient counseling, and closer postoperative surveillance, while in elective settings low SMG may also help identify patients who could benefit from preoperative optimization.

## 2. Materials and Methods

### 2.1. Study Design and Setting

This retrospective, single-center observational study was conducted at the Second Clinic of Surgery, Emergency County Clinical Hospital of Târgu Mureș, Romania. Consecutive adult patients undergoing major colorectal cancer surgery and admitted between January 2022 and November 2025 were screened for eligibility. The study was approved by the institutional ethics committee (approval number: 21914, date: 10 September 2025), and patient data were analyzed in anonymized form in accordance with institutional and applicable regulatory requirements. This study is reported in accordance with the Strengthening the Reporting of Observational Studies in Epidemiology (STROBE) guidelines.

### 2.2. Study Population

All potentially CRC-related admissions during the study period (January 2022–November 2025) were screened using relevant ICD codes (D37.x, C18–C20, K56.4). Patients were eligible if they met the following inclusion criteria: (i) age ≥ 18 years; (ii) histologically confirmed primary colorectal cancer; (iii) underwent colorectal surgery at our institution; (iv) availability of a preoperative, native (non-contrast) abdominopelvic CT suitable for body composition analysis; and (v) complete 30-day postoperative follow-up data for the study outcomes.

**Figure 1:**
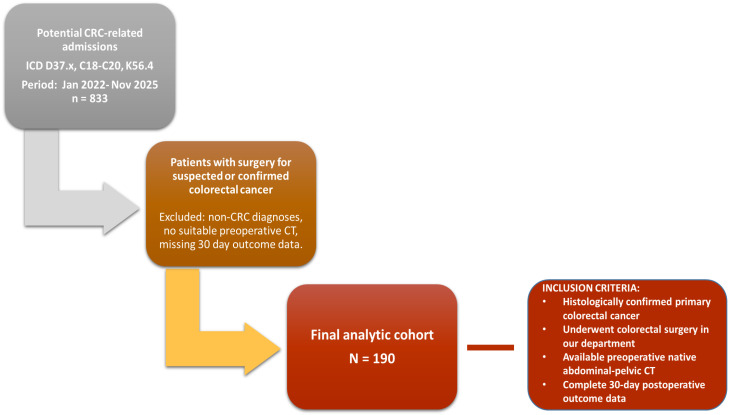
Flowchart of the patient selection and final cohort.

Exclusion criteria were: (i) absence of a suitable preoperative CT examination; (ii) non-operative management or no colorectal surgical treatment performed; (iii) alternative (non-CRC) final diagnosis or insufficient documentation to confirm the diagnosis; and (iv) missing essential perioperative or outcome data, including variables required to classify the surgical procedure and to ascertain 30-day postoperative outcomes. The final analytic cohort comprised 190 patients (Figure 1). The cohort therefore reflects a real-world institutional CRC surgical population including both elective and emergency presentations.

### 2.3. Data Collection and Variable Definitions

Clinical data were retrospectively extracted from archived patient records and the institutional electronic hospital database (H3 Healthcare Concept). Imaging data were retrieved from the local Picture Archiving and Communication System (PACS), and operative details were obtained from operative reports and standardized surgical protocols. For each eligible patient, the following variables were collected and defined according to prespecified definitions.

Demographics, anthropometrics, and admission characteristics: Age and sex were recorded at admission. Body weight and height were extracted from admission documentation, and body mass index (BMI) was calculated as weight (kg) divided by height squared (m^2^). Mode of admission was categorized as elective or emergency, with emergency admission defined as presentation through the Emergency Department. In addition, a separate urgency variable captured whether surgery was performed within 24 h of admission (immediate surgery). Presentation severity was further characterized by the presence of obstructive and/or perforative disease (e.g., ileus and/or perforation), based on clinical, radiological, and operative documentation.

Tumor and disease characteristics: Tumor location was categorized as right colon (cecum, ascending colon, hepatic flexure, and transverse colon), left colon (splenic flexure, descending colon, and sigmoid colon), or rectum. Local organ invasion was recorded as a binary variable (yes/no), defined as tumor invasion or direct extension into adjacent organs or structures. In addition, the presence of metastatic disease at presentation was documented (yes/no), based on imaging, operative findings, and/or histopathology where available.

Surgical and perioperative variables: Operative approach (open versus laparoscopic, including conversions where applicable) and procedural details were recorded, including whether a colorectal resection was performed (yes/no), creation of a stoma, performance of an anastomosis, and anastomotic configuration/type when documented in operative reports.

Outcomes: Postoperative outcomes included length of hospital stay (LOS), intensive care unit (ICU) admission (yes/no), and ICU length of stay (ICU days). Postoperative morbidity was summarized using the Comprehensive Complication Index (CCI). The primary outcome was 30-day major complications (major events), defined as Clavien–Dindo grade ≥ IIIb. Reoperation and readmission within 30 days were recorded. Additional clinically relevant events included perioperative blood transfusion, postoperative vasopressor requirement (defined as any vasopressor infusion administered postoperatively), initiation of hemodialysis, and 30-day mortality.

Laboratory data: Preoperative laboratory values were extracted from routine blood tests performed within 1–2 days before surgery. Collected parameters included complete blood count (including hemoglobin) and inflammatory/nutritional markers, including C-reactive protein (CRP), serum albumin, and total protein. These laboratory variables were used to calculate the pan-immune-inflammation value (PIV) as described below.

### 2.4. Outcomes

The primary outcome was the occurrence of 30-day major postoperative complications, defined as Clavien–Dindo grade ≥ IIIb within 30 days after surgery. Deaths within 30 days were classified as Clavien–Dindo grade V and were therefore included in the primary endpoint; 30-day mortality was additionally recorded and reported as a separate outcome. Secondary outcomes included overall postoperative morbidity quantified by the Comprehensive Complication Index (CCI), length of hospital stay (LOS), ICU admission (yes/no), and ICU length of stay (ICU days). Additional 30-day outcomes recorded were reoperation, readmission, postoperative vasopressor requirement, hemodialysis, and perioperative blood transfusion.

### 2.5. CT-Derived Body Composition and SMG Assessment

Preoperative abdominopelvic CT examinations were analyzed using the non-contrast (native) phase. Skeletal muscle assessment was performed at the third lumbar vertebra (L3) on an axial slice at the level of the transverse processes, consistent with established body composition methodology. Image segmentation was conducted using 3D Slicer (version 5.8.1, open-source software), and skeletal muscle tissue was identified using predefined attenuation thresholds of −30 to +150 Hounsfield units (HU).

The skeletal muscle area (SMA, cm^2^) was calculated as the cross-sectional area of the following muscles at the L3 level: rectus abdominis; transversus abdominis; external and internal oblique muscles; psoas major; quadratus lumborum; erector spinae muscle group (including iliocostalis, longissimus, and spinalis); and deep paraspinal muscles. The skeletal muscle index (SMI, cm^2^/m^2^) was calculated as SMA divided by height squared (m^2^). Mean skeletal muscle attenuation (HU) was recorded from the segmented muscle region and used as an indicator of muscle quality/myosteatosis. Skeletal muscle gauge (SMG) was calculated as SMI × mean muscle attenuation (HU), thereby integrating muscle quantity and muscle quality in a single composite metric.

### 2.6. Pan-Immune-Inflammation Value

Preoperative PIV was calculated from routine blood tests obtained within 1–2 days before surgery using the formula: PIV = (neutrophil count × platelet count × monocyte count)/lymphocyte count. Cell counts were recorded from the complete blood count and expressed in consistent units as reported by the institutional laboratory. For patients with multiple preoperative measurements within the defined window, the value closest to the time of surgery was used. Cases with missing components required for PIV calculation were excluded from PIV-based analyses.

### 2.7. Phenotype Classification

Receiver operating characteristic (ROC) curve analyses were performed to evaluate the ability of SMG and PIV to discriminate patients with and without 30-day major complications. Optimal cut-off values for each marker were determined using the Youden index (J = sensitivity + specificity − 1). Based on these thresholds, SMG and PIV were dichotomized into binary variables: SMG_low (coded 1 for SMG below the cut-off; 0 otherwise) and PIV_high (coded 1 for PIV at or above the cut-off; 0 otherwise).

Using the combination of SMG_low and PIV_high, patients were classified into four phenotypes: (1) low PIV/high SMG (reference “healthiest” phenotype), (2) low PIV/low SMG, (3) high PIV/high SMG, and (4) high PIV/low SMG, the latter representing the biologically least favorable combined phenotype.

### 2.8. Statistical Analysis

The primary endpoint was the occurrence of 30-day major postoperative complications, defined as any complication with Clavien–Dindo grade ≥ IIIb. Baseline characteristics (Table 1) were summarized for patients with and without 30-day major complications. Continuous variables are presented as mean ± SD when approximately normally distributed (e.g., mean muscle attenuation) and were compared using the independent-samples Student’s *t*-test; Levene’s test assessed homogeneity of variances, and the Welch *t*-test was used when variances were unequal. Non-normally distributed continuous variables are reported as median (IQR) and were compared using the Mann–Whitney U test. Categorical variables are presented as n (%) and were compared using the chi-square test or Fisher’s exact test, as appropriate.

To explore associations between candidate predictors and major complications, univariable logistic regression analyses were performed, reporting odds ratios (ORs) with 95% confidence intervals (CIs). For multivariable analysis, we constructed a prespecified parsimonious logistic regression model to minimize overfitting given the limited number of events. The model included clinically relevant covariates (age, modeled per 10-year increase; surgical urgency; procedure type) and the key exposures of interest (low SMG and high PIV). All variables were entered simultaneously using the Enter method. Given the limited number of major events, the adjusted model was intentionally kept parsimonious to reduce the risk of overfitting. Additional laboratory variables that differed descriptively between groups, such as CRP, albumin, and total protein, were not included in the primary multivariable model because they showed substantial variable-specific missingness, and their inclusion would have markedly reduced the analyzable sample and compromised model stability. The primary adjusted model was therefore restricted to clinically relevant perioperative variables with broader availability; however, residual confounding cannot be excluded. Model calibration was assessed using the Hosmer–Lemeshow goodness-of-fit test, and explanatory performance was summarized with Nagelkerke R^2^. Statistical significance was set at a two-sided *p* < 0.05.

For descriptive analyses, dichotomized SMG and PIV were combined into four preoperative host phenotypes: (1) low PIV with normal SMG, (2) low PIV with low SMG, (3) high PIV with normal SMG, and (4) high PIV with low SMG. The proportions of patients with 30-day major complications and ICU admission were compared across phenotypes using the chi-square test (with linear-by-linear association as a test for trend). The Comprehensive Complication Index (CCI) and length of hospital stay (LOS) were compared across phenotypes using the Kruskal–Wallis test, and are reported as medians (IQRs).

Discriminative ability of SMG, PIV, and multivariable models for predicting 30-day major complications was evaluated using receiver operating characteristic (ROC) curve analysis. Area under the curve (AUC) values with 95% CIs were obtained using the non-parametric ROC method. Optimal cut-offs for SMG and PIV were identified by maximizing Youden’s index (sensitivity + specificity − 1) and were used to define low SMG and high PIV in the main analyses. To assess incremental discriminative value beyond clinical variables, three logistic regression models were evaluated: (1) a clinical model (age, surgical urgency, procedure type), (2) the clinical model plus low SMG, and (3) the clinical model plus low SMG and high PIV. Predicted probabilities from each model were used as test variables in ROC analyses; differences in AUCs were interpreted descriptively based on point estimates and confidence intervals.

Analyses were performed in IBM SPSS Statistics (version 25; IBM Corp., Armonk, NY, USA). Unless otherwise stated, analyses were conducted on an available-case basis; therefore, denominators vary according to variable-specific completeness. For key preoperative laboratory variables, complete blood count components used for PIV calculation were available in 178/190 patients (93.7%), hemoglobin in 179/190 (94.2%), CRP in 88/190 (46.3%), total protein in 55/190 (28.9%), and albumin in 44/190 patients (23.2%). No imputation was performed. No formal sensitivity analysis for missing data was undertaken.

## 3. Results

### 3.1. Baseline Demographic, Clinical, and Operative Characteristics

In total, 190 patients undergoing major colorectal cancer surgery were included in the final analytic cohort. Baseline demographic, clinical, laboratory, and perioperative characteristics stratified by 30-day major complications are summarized in Table 1.

**Table 1:** Clinical, Laboratory, and Perioperative Characteristics Stratified by 30-Day Major Complications.

	Variable	N (Valid)	NME (Summary)	N NME (Valid)	ME (Summary)	N ME (Valid)	*p* Value
Demographics							
	AGE (years)	190	70 (63–76)	137	72 (66–81)	53	0.045
	BMI (kg/m^2^)	190	26.6 (23.4–29.3)	137	24.9 (21.7–29.4)	53	0.245
	SEX (male, n (%))	190	79 (57.7%)	137	29 (54.7%)	53	0.713
Tumor location		190		137		53	0.118
	Right Colon n (%)		46 (33.6%)		22 (41.5%)		
	Left Colon n (%)		64 (46.7%)		27 (50.9%)		
	Rectum n (%)		27 (19.7%)		4 (7.5%)		
Surgical characteristics							
	Urgency (emergency, n (%))	190	73 (53.3%)	137	46 (86.8%)	53	<0.001
	Bowel obstruction n (%)	190	56 (40.9%)	137	33 (62.3%)	53	0.008
	Perforation/abscess n (%)	190	12 (8.8%)	137	16 (30.2%)	53	<0.001
	Metastasis/Penetration n (%)	190	49 (35.8)	137	25 (47.2)	53	0.148
	Immediate operation n (%)	190	56 (40.9%)	137	36 (67.9%)	53	0.001
	Resection n (%)	190	125 (91.2)	137	42 (79.2)	53	0.023
	Stoma n (%)	190	66 (48.2)	137	34 (64.2)	53	0.048
	Anastomosis n (%)	190	72 (52.6)	137	20 (37.7)	53	0.067
	Stapled anastomosis n (%)	91	35 (47.9)	73	7 (38.9)	18	0.49
	Laparotomy	190	117 (85.4)	137	49 (92.5)	53	0.189
Laboratory							
	WBC	178	8.10 (6.08–10.67)	126	9.96 (7.66–14.96)	51	0.001
	NEU	178	5.86 (4.10–8.15)	127	7.68 (5.3–12.79)	51	0.003
	LYM	178	1.23 (1.02–1.78)	127	1.11 (0.79–1.73)	51	0.156
	PLT	178	317 (229–386)	127	303 (206–374)	51	0.494
	MONO	178	0.625 (0.467–0.820)	126	0.690 (0.440–0.990)	51	0.459
	Protein	55	6.88 (6.25–7.23)	42	5.76 (5.03–6.24)	13	0.003
	Albumin	44	4.11 (3.88–4.33)	34	3.02 (2.56–3.63)	10	0.001
	CRP	88	8.4 (2.39–64.54)	61	82.4 (27.5–138)	27	0.001
	HB (g/dL)	179	11.9 (10.22–13.4)	128	11 (9.9–13.4)	51	0.283
	PIV	178	944.18 (413.16–1641.10)	127	1684 (512.44–4026.24)	51	0.047
	PIV category (≥1680.2, n (%))	178	29 (22.8)	127	26 (51)	51	<0.001
	Low P/A (low, n (%))		9 (16.1)	56	11 (61.1)	18	<0.001
CT-derived body composition							
	SMI area/m^2^	190	40.90 (34.52–47.85)	137	33.69 (28.77–39.72)	53	<0.001
	SMD HU mean (±SD)	190	30.76 (±8.62)	137	24.80 (±9.86)	53	<0.001
	SMG	190	1222.94 (934.59–1594.45)	137	780.55 (462.44–1226.34)	53	<0.001
	low SMG (SMG ≤ 867.88, n (%))	190	26 (19)	137	32 (60.4)	53	<0.001
Outcomes							
	LOS days	190	9 (8–11)	137	12 (4.5–24)	53	0.076
	ICU (yes, n (%))	190	18 (13.1)	137	42 (79.2)	53	<0.001
	ICU days	190	0 (0–0)	137	2 (1–6)	53	<0.001
	30-day mortality (n (%))	190	0 (0)	137	30 (56.6)	53	<0.001
	CCI	190	0 (0–8.7)	137	100 (42.4–100)	53	<0.001

Continuous variables are presented as median (IQR); categorical variables as n (%). *p*-values from Mann–Whitney U and Student T test for continuous variables and χ^2^ test for categorical variables. NME—no major event, ME—major event, BMI—body mass index, WBC—white blood cell, NEU—neutrophil, LYM—lymphocyte, PLT—platelet, MONO—monocyte, CRP—C-reactive protein, HB—hemoglobin, PIV—pan-immune-inflammation index, Low P\A—low protein or albumin, SMI—skeletal muscle index, SMD—skeletal muscle density, SMG—skeletal muscle gauge, LOS—length of stay, ICU—intensive care unit, CCI—Comprehensive Complication Index.

Patients with 30-day major complications were older than those without major events (median 72 vs. 70 years; *p* = 0.045), while sex and BMI did not differ significantly between groups. Major events were more frequent in emergency and immediately operated patients and were associated with bowel obstruction/perforation, higher inflammatory burden (including WBC/CRP and PIV), and a markedly adverse body-composition profile (lower SMA, lower mean muscle attenuation, and lower SMG; all *p* < 0.001). Clinically, the major-complication group had substantially higher ICU utilization and mortality and a markedly increased CCI (all *p* < 0.001), whereas LOS showed only a nonsignificant trend toward longer hospitalization.

### 3.2. Predictors of 30-Day Major Postoperative Complications

In univariable logistic regression (Table 2), higher age, emergency surgery, non-resection procedures, low SMG, and high PIV were all associated with an increased risk of 30-day major complications. The odds of a major complication increased by 38% per 10-year increase in age (OR 1.38, 95% CI 1.01–1.87). Emergency surgery was associated with 5.76-fold higher odds of major complications compared with elective surgery (95% CI 2.43–13.66), and non-resection/diverting operations were associated with higher odds than resection procedures (OR for resection vs. non-resection 0.36, 95% CI 0.15–0.89). Low SMG was strongly associated with major complications (OR 6.50, 95% CI 3.24–13.05), and high PIV was likewise associated with increased odds (OR 3.51, 95% CI 1.77–6.99).

In the multivariable model including age, surgical urgency, procedure type, low SMG, and high PIV (Table 2), emergency surgery and low SMG remained independent predictors of 30-day major complications. Emergency procedures were associated with approximately 3.5-fold higher odds of major complications compared with elective surgery (adjusted OR 3.48, 95% CI 1.32–9.14), while low SMG was associated with more than four-fold higher odds (adjusted OR 4.17, 95% CI 1.91–9.09). High PIV retained a positive association with major complications, but this did not retain conventional statistical significance after adjustment (adjusted OR 2.05, 95% CI 0.91–4.58). Age (per 10-year increase) and procedure type (resection vs. non-resection) were not independently associated with major complications in the adjusted model.

### 3.3. SMG–PIV Host Phenotypes and Postoperative Outcomes

When SMG and PIV were combined into four preoperative host phenotypes (Table 3), marked gradients in complication burden and ICU use were observed. The most favorable phenotype (low PIV/normal SMG) comprised 90 patients (47.4%) and had a 30-day major complication rate of 14.4% (13/90). In contrast, patients with low PIV but low SMG had a higher rate of major events (36.4%, 12/33), and those with high PIV but preserved SMG had an intermediate risk (25.0%, 8/32). The least favorable phenotype (high PIV/low SMG, n = 23) experienced major complications in 78.3% of cases (18/23) (overall chi-square *p* < 0.001).

**Figure 2:**
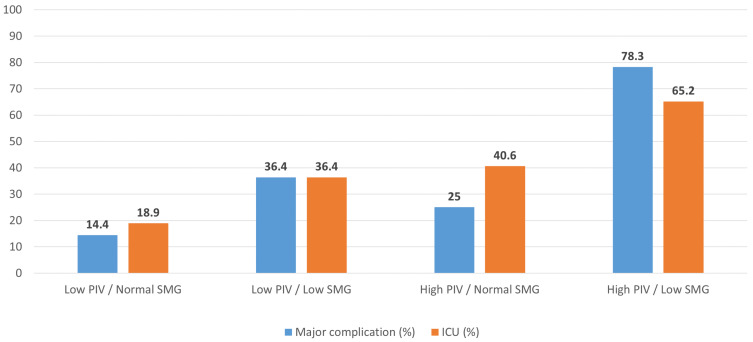
Thirty-day major complications and ICU admission by SMG–PIV host phenotype. Bars show the percentage of patients with 30-day major complications (Clavien–Dindo ≥ IIIb) and ICU admission in each phenotype group: (1) low PIV/normal SMG (n = 90), (2) low PIV/low SMG (n = 33), (3) high PIV/normal SMG (n = 32), and (4) high PIV/low SMG (n = 23). SMG and PIV were dichotomized using ROC/Youden-derived cut-offs.

A similar pattern was seen for the overall postoperative burden. Median CCI increased progressively from 0.0 [0.0–20.9] in the low PIV/normal SMG group to 12.2 [0.0–49.6] in low PIV/low SMG, 20.9 [0.0–24.2] in high PIV/normal SMG and 100 [22.6–100] in high PIV/low SMG (Kruskal–Wallis *p* < 0.001). In contrast, LOS did not differ significantly between phenotypes (similar medians, respectively; *p* = 0.793). ICU admission became progressively more frequent from the most to the least favorable profile, occurring in 18.9% of patients with low PIV/normal SMG, 36.4% with low PIV/low SMG, 40.6% with high PIV/normal SMG and 65.2% with high PIV/low SMG (chi-square *p* < 0.001). These differences in major complication and ICU admission rates across phenotypes are also illustrated in Figure 2.

### 3.4. Discriminative Performance and ROC Curve Analysis

In ROC analysis (Table 4), SMG as a continuous variable showed moderate discriminative ability for 30-day major complications, with an AUC of 0.732 (95% CI 0.647–0.816). The optimal SMG threshold identified by Youden’s index was 867.9 AU, yielding a sensitivity of 60.4% and a specificity of 81.0%. In contrast, PIV alone showed limited discriminative ability, with an AUC of 0.595 (95% CI 0.491–0.700) and a Youden-derived cut-off of 1680.2, corresponding to a sensitivity of 51.0% and a specificity of 77.2%. The lower bound of the CI approached 0.50, indicating that PIV by itself discriminated major complications only slightly better than chance.

**Figure 3:**
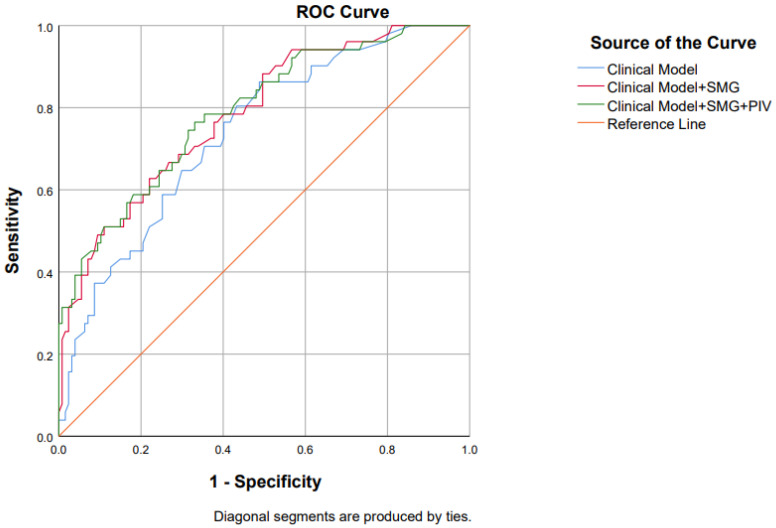
ROC curves for predicting 30-day major postoperative complications (Clavien–Dindo ≥ IIIb) using the clinical model (age, emergency surgery, and procedure type) and the clinical model extended with low SMG and high PIV. Curves were generated from predicted probabilities of each logistic regression model; SMG—skeletal muscle gauge, PIV—pan-immune-inflammation value.

The clinical model including age, surgical urgency and procedure type achieved an AUC of 0.739. Adding low SMG to this model improved the AUC to 0.784, indicating a moderate gain in discriminative performance. Further addition of high PIV resulted in a small additional increase in AUC to 0.791. Although the confidence intervals of these AUCs overlapped, these findings suggest that SMG provides relevant incremental discriminative value beyond basic clinical variables, whereas the additional contribution of PIV was modest. These differences in discriminative performance are illustrated by the ROC curves shown in Figure 3.

## 4. Discussion

In this retrospective single-center cohort of patients undergoing major colorectal cancer surgery, low preoperative skeletal muscle gauge (SMG) was strongly associated with 30-day major complications and remained independently associated after adjustment for age, surgical urgency, procedure type, and high pan-immune-inflammation value (PIV). High PIV was also associated with major complications in univariable analysis, but its effect attenuated after adjustment. When combined, SMG and PIV identified a host phenotype characterized by markedly increased major complication rates and ICU use. In ROC analyses, SMG provided the main incremental discriminative value beyond basic clinical variables, whereas PIV contributed only a modest additional improvement.

In this study, we interpreted early postoperative morbidity after colorectal cancer surgery through an integrated host-phenotype framework combining body composition and immune–inflammatory burden. Reduced skeletal muscle quantity and quality may reflect diminished physiologic reserve, as skeletal muscle serves as the body’s largest protein reservoir and provides amino acids required during major surgical stress to support immune function and tissue repair [21,22]. In patients with sarcopenia and myosteatosis, depletion of these reserves may reduce tolerance to surgical stress and impair postoperative recovery [23]. In parallel, heightened systemic inflammation—whether related to tumor biology, cancer-associated cachexia, or acute clinical severity—may amplify catabolic pathways, worsen muscle depletion, and disrupt metabolic homeostasis [24]. Importantly, an elevated inflammatory signal may also act as a surrogate for adverse local disease states such as obstruction, perforation, necrosis, or infection, which themselves increase perioperative risk [25,26]. Taken together, these interconnected nutritional and inflammatory mechanisms provide a biologically plausible rationale for evaluating body composition parameters alongside inflammation-based indices when identifying patients at risk for severe postoperative morbidity.

CT-based body composition measures that capture both muscle quantity and quality have been consistently linked to early postoperative outcomes after colorectal cancer surgery [27,28,29], which is consistent with the rationale for skeletal muscle gauge (SMG) as a composite metric integrating SMI and mean muscle attenuation. In a cohort of patients undergoing open colon resection for cancer, Boer et al. showed that mean muscle attenuation (HU)—a proxy of muscle quality/myosteatosis—was significantly lower in patients who developed postoperative complications (e.g., L3 HU of total abdominal muscle area 37.1 ± 10.0 vs. 29.8 ± 8.8; *p* < 0.001) and remained independently associated with overall complications in multivariable models (OR per 1-HU increase ≈0.91–0.92 across levels; *p* ≤ 0.003) [30].

Chai et al. (2021) reported a significantly higher major complication rate in sarcopenic patients (11.0% vs. 6.7%), a higher Comprehensive Complication Index (CCI) score (*p* = 0.002), a 7.4-day longer length of stay (LOS), higher 30-day mortality (4.7% vs. 0.8%), and a higher readmission rate (13.4% vs. 4.5%) [31]. Ölmez et al., using CT-derived skeletal muscle index (SMI), found higher rates of Clavien–Dindo ≥ III complications (17.8% vs. 5.7%) and a longer ICU stay (*p* = 0.002). Similar to our findings, the difference in LOS between low and normal SMI patients was not significant [32]. Overall, several studies using qualitative and/or quantitative assessment of skeletal muscle have demonstrated worse early postoperative outcomes in sarcopenic CRC patients [33,34,35]. However, studies specifically evaluating SMG in relation to early postoperative complications after CRC surgery remain uncommon. In a different surgical setting, Evans RPT et al. demonstrated the value of preoperative stratification using SMG before emergency laparotomy; patients with low SMG had higher 30-day mortality (multivariable OR 2.12, 95% CI 1.05–4.29; *p* = 0.037) and longer LOS (*p* < 0.001) [36]. SMG has also been associated with early postoperative risk in other types of surgery; for example, Sanderfer et al. reported similar associations in esophagectomy cohorts [37].

More broadly, contemporary colorectal cancer literature synthesized in recent reviews supports that sarcopenia and related CT phenotypes are associated not only with higher postoperative morbidity but also with downstream “nutrition-relevant” consequences such as delayed recovery and increased resource utilization (e.g., prolonged stay and critical care needs), reinforcing the rationale for composite indices such as SMG when studying perioperative risk in a nutrition-focused oncology context [35,38,39].

Complementing imaging-derived assessment, inflammation-based indices derived from routine blood counts have gained attention as pragmatic markers of host response. PIV integrates neutrophils, platelets, monocytes, and lymphocytes into a single composite measure and has been proposed to reflect immune–inflammatory dysregulation more comprehensively than simpler ratios [19,39]. In our cohort, high PIV was associated with adverse postoperative outcomes in unadjusted analyses and contributed to risk separation when combined with SMG in the phenotype framework; however, its association attenuated in the parsimonious multivariable model that included age, urgency, and procedure type. This pattern suggests that PIV may partly capture acute clinical severity and operative context already represented by these key clinical variables. Accordingly, in this setting PIV appears to provide complementary rather than independent perioperative information, particularly when interpreted alongside CT-derived muscle phenotype.

Similar to our findings, Seo et al. reported that patients with high PIV had a higher rate of postoperative complications than those with low PIV and identified PIV as an independent predictor in multivariable analysis (adjusted OR 1.92). In our cohort, high PIV was also associated with major complications in univariable analysis (OR 3.51, *p* < 0.001); however, after adjustment, the association attenuated and did not reach statistical significance (adjusted OR 2.05, *p* = 0.082). Despite these differences in statistical significance, the adjusted effect sizes were comparable, suggesting a broadly consistent direction and magnitude of association across cohorts. ROC performance was modest in both studies (AUC 0.618 in Seo et al. and 0.595 in our cohort), indicating limited discriminative ability when PIV is used as a standalone marker [40]. However, when PIV was combined with SMG and the clinical model, overall discrimination increased modestly to an AUC of 0.791. Collectively, these findings support the interpretation that PIV may provide complementary value for identifying higher-risk patients rather than serving as a standalone biomarker.

Most recently published CRC studies evaluating PIV have focused primarily on long-term oncologic outcomes rather than early postoperative morbidity and mortality [39,41]. Accordingly, additional work is warranted to better define the perioperative utility of PIV and to clarify the contexts in which it provides incremental value beyond clinical variables and body composition. To our knowledge, no prior study has combined PIV and SMG to define preoperative host phenotypes for risk stratification in CRC surgical patients.

According to our results, the high PIV/low SMG phenotype identifies a subgroup with particularly high postoperative burden, including 30-day major complications and ICU use. However, the clinical implications of this phenotype differ by operative setting. In elective patients, low SMG may represent a potentially modifiable target, and referral for nutrition-focused optimization and structured prehabilitation may be reasonable when a meaningful preoperative window exists. In contrast, in urgent or emergency presentations the phenotype is less useful as a target for preoperative modification and more relevant as a marker of heightened perioperative vulnerability. In that setting, its practical value lies primarily in supporting early recognition of patients who may require closer postoperative monitoring and escalation readiness [42,43,44]. Unlike low SMG—which may represent a potentially modifiable target in elective patients—an isolated high PIV is not itself a directly treatable preoperative target, but rather a marker of heightened immune–inflammatory burden [19,45,46]. In practical perioperative workflows, this combined information may support risk-adapted ERAS planning, prioritization for prehabilitation or dietitian referral in elective cases, and more informed surgical decision-making in borderline candidates in whom physiologic reserve is a relevant concern.

In the emergency setting, where there is no time for meaningful prehabilitation, patients with the low SMG/high PIV phenotype should be considered a high-priority group for intensified postoperative surveillance. This includes a lower threshold for early escalation (e.g., sepsis/leak workup), closer hemodynamic monitoring, and proactive implementation of ERAS-compatible recovery measures with early nutrition support. In practical terms, the phenotype can guide triage of monitoring intensity and resource allocation during the early postoperative period.

This study has several limitations. First, the retrospective, single-center design introduces inherent risks of selection bias, residual confounding, and limited generalizability. Second, our cohort included a high proportion of urgent and complicated presentations, including emergency surgery, bowel obstruction, and perforation/abscess, which likely amplified both inflammatory burden and postoperative event rates. As a result, the observed associations, particularly those related to PIV, may partly reflect acute disease severity and should not be generalized directly to predominantly elective CRC surgical populations. Future studies with larger cohorts should evaluate these associations separately in elective and emergency colorectal cancer populations. This inclusion of both elective and emergency cases introduced additional clinical heterogeneity into the cohort and the predictive model was not externally validated and therefore requires confirmation in independent cohorts before broader clinical application. Third, although the cohort size was adequate for the primary analyses, the moderate sample and event counts constrained model complexity and limited the ability to perform robust subgroup and sensitivity analyses. Fourth, we did not systematically account for preoperative oncologic treatments (e.g., chemotherapy and/or radiotherapy), which may influence inflammatory status and body composition and could therefore confound observed associations. Fifth, because structured prehabilitation pathways are not routinely implemented in our institution, we could not evaluate whether targeted nutritional or exercise-based optimization modifies risk in the identified phenotypes. Sixth, several laboratory variables, particularly CRP, total protein, and albumin, had substantial missingness, which limited their use in adjusted analyses and may have contributed to residual confounding. Finally, the SMG cut-off was derived from ROC analysis in the overall study cohort and was not sex-specific or externally validated. Therefore, this threshold should be interpreted as cohort-specific and hypothesis-generating, and future studies should validate sex-specific and externally applicable cut-offs in independent cohorts to improve generalizability and clinical interpretability.

## 5. Conclusions

Preoperative risk assessment before major colorectal cancer surgery may be strengthened by identifying patients with low skeletal muscle gauge (SMG), particularly those with the combined low SMG/high PIV phenotype, to support prioritization of perioperative resources and intensified postoperative surveillance. Low SMG emerged as the more robust preoperative marker in this cohort, while PIV provided complementary rather than independent information within the combined phenotype framework. In elective settings, low SMG may represent a potentially modifiable target for nutrition-focused optimization and prehabilitation when a meaningful preoperative window exists. Prospective multicenter studies are warranted to validate these cut-offs and determine whether phenotype-guided optimization improves clinical outcomes.

## Figures and Tables

**Table 2:** Predictors of 30-Day Major Postoperative Complications: Univariable and Multivariable Logistic Regression.

Predictor	Univariable OR (95% Cl)	Univariable *p*	Multivariable OR (95% Cl)	Multivariable *p*
Age, per 10-year increase (age10)	1.38 (1.01–1.87)	0.043	1.27 (0.89–1.83)	0.193
Emergency surgery (yes vs. elective)	5.76 (2.43–13.66)	<0.001	3.48 (1.32–9.14)	0.012
Resection procedure (vs. non-resection)	0.36 (0.15–0.89)	0.027	0.52 (0.17–1.53)	0.233
Low SMG (yes vs. no)	6.5 (3.24–13.05)	<0.001	4.17 (1.91–9.09)	<0.001
High PIV (yes vs. no)	3.51 (1.77–6.99)	<0.001	2.05 (0.91–4.58)	0.082

Adjusted for age, surgical urgency, procedure type, low SMG and high PIV in the same model. Non-resection procedures include diverting or palliative operations such as loop colostomy ± biopsy only; resection procedures include segmental colectomy, anterior resection, Hartmann’s procedure, etc. OR—odds ratio, SMG—skeletal muscle gauge, PIV—pan-immune-inflammation value.

**Table 3:** Thirty-Day Major Complications and Secondary Outcomes across Combined SMG–PIV Phenotypes.

Phenotype Group	n	NME n (%)	ME n (%)	CCI Median (IQR)	LOS Median (IQR)	No ICU n (%)	ICU n (%)
1	90	77 (85.6)	13 (14.4)	0.0 (0.0–20.9)	9 (7.75–11.25)	73 (81.1)	17 (18.9)
2	33	21 (63.6)	12 (36.4)	12.2 (0.0–49.55)	9 (7.5–12)	21 (63.3)	12 (36.4)
3	32	24 (75)	8 (25)	20.9 (0.0–24.2)	9 (8–13)	19 (59.4)	13 (40.6)
4	23	5 (21.7)	18 (78.3)	100 (22.6–100)	11 (6–16)	8 (34.8)	15 (65.2)

NME—no major event, ME—major event, CCI—Comprehensive Complication Index, LOS—length of stay, ICU—intensive care unit, Chi-square test for ME *p* < 0.001, Kruskal–Wallis test for CCI *p* < 0.001, Kruskal–Wallis test for LOS *p* = 0.793, Chi-square test for ICU *p* < 0.001, 1 = low PIV/normal SMG, 2 = low PIV/low SMG, 3 = high PIV/normal SMG, 4 = high PIV/low SMG.

**Table 4:** Discriminative Performance of SMG, PIV, and Multivariable Models for Predicting 30-Day Major Complications.

Predictor/Model	AUC (95% CI)	Optimal Cut-Off	Sensitivity (%)	Specificity (%)
SMG continuous	0.732 (0.647–0.816)	867.88	60.4	81
PIV continuous	0.595 (0.491–0.700)	1680.19	51	77.2
Clinical model (age, surgical urgency, procedure type)	0.739 (0.661–0.816)			
Clinical model + SMG	0.784 (0.711–0.857)			
Clinical model + SMG + PIV	0.791 (0.717–0.864)			

Optimal cut-offs were derived by maximizing Youden’s index and were used to define low SMG and high PIV. The clinical model included age, surgical urgency (emergency surgery yes/no), and procedure type (resection of the primary tumor vs. non-resection/palliative procedures, including diverting stoma, internal bypass/derivation, or biopsy-only). AUCs are reported with 95% confidence intervals.

## Data Availability

The data presented in this study are available on reasonable request from the corresponding author.

## Abbreviations

The following abbreviations are used in this manuscript:

SMG	Skeletal Muscle Gauge
PIV	Pan-Immune-Inflammation Value
CRC	Colorectal cancer
OR	Odds ratio
CI	Confidence interval
AUC	Area under the curve
CT	Computed tomography
STROBE	Strengthening the Reporting of Observational Studies in Epidemiology
ICD	International Classification of Diseases
PACS	Picture Archiving and Communication System
BMI	Body mass index
LOS	Length of stay
ICU	Intensive care unit
CCI	Comprehensive Complication Index
CRP	C-reactive protein
HU	Hounsfield Units
L3	3rd Lumbar vertebra
SMA	Skeletal Muscle Area
SMI	Skeletal Muscle Index
ROC	Receiver Operating Characteristic
IQR	Interquartile range
SD	Standard deviation
NME	No major event
ME	Major event
WBC	White blood cell
NEU	Neutrophil
LYM	Lymphocyte
PLT	Platelet
MONO	Monocyte
HB	Hemoglobin
